# Genome Sequence Comparison of Staphylococcus aureus TX0117 and a Beta-Lactamase-Cured Derivative Shows Increased Cationic Peptide Resistance Accompanying Mutations in *relA* and *mnaA*

**DOI:** 10.1128/MRA.01515-19

**Published:** 2020-04-30

**Authors:** Mia Jade Sales, George Sakoulas, Richard Szubin, Bernhard Palsson, Cesar Arias, Kavindra V. Singh, Barbara E. Murray, Jonathan M. Monk

**Affiliations:** a Bioengineering Department, University of California San Diego, La Jolla, California, USA; b Department of Pediatrics, University of California, San Diego School of Medicine, La Jolla, California, USA; c Division of Infectious Diseases, Department of Internal Medicine, McGovern Medical School at the University of Texas Health Science Center at Houston (UTHealth), Houston, Texas, USA; d Center for Antimicrobial Resistance and Microbial Genomics, McGovern Medical School at the University of Texas Health Science Center at Houston (UTHealth), Houston, Texas, USA; Indiana University, Bloomington

## Abstract

Staphylococcus aureus strain TX0117 is a methicillin-susceptible bacterium with type A beta-lactamase exhibiting a high cefazolin inoculum effect. TX0117 was cured of *blaZ*, yielding TX0117c with increased antimicrobial peptide resistance. The sequencing and genome assembly of TX0117 elucidate six mutations between TX0117 and TX0117c, including *relA* truncation and *mnA_1* substitution.

## ANNOUNCEMENT

Beta-lactam therapy has been associated with better outcomes than non-beta-lactam therapy (i.e., vancomycin) in patients with methicillin-susceptible Staphylococcus aureus (MSSA) bacteremia (1–4). Treatment standards recommend therapy with either an isoxazolyl penicillin (e.g., nafcillin, oxacillin) or cefazolin (5). Recent data have emerged to show that cefazolin demonstrates similar efficacy but better tolerability in patients than do antistaphylococcal isoxazolyl penicillins (6, 7). However, cefazolin treatment failures have been reported due to a cefazolin inoculum effect, which is defined by isolates showing an MIC of ≥16 mg/liter in assays utilizing a bacterial inoculum of 10^7^ CFU/ml compared to the standard inoculum of 10^5^ CFU/ml (8–11). The cefazolin inoculum effect is based on the ability of the beta-lactamase of some MSSA strains to overcome and hydrolyze cefazolin when bacteria are at high inoculum, and it has been shown to cause clinical failures in certain deep-seated infections. These isolates may be uncommon, but considerable regional variability is seen in their prevalence (12–15).

In order to examine the effects of different antimicrobial therapies *in vitro* against MSSA with a significant cefazolin inoculum effect, a clinical strain was isolated from a patient with MSSA endocarditis who relapsed after cefazolin therapy (strain TX0117) (11). This strain was subsequently cured by heat at 43°C and by novobiocin exposure to inactivate the beta-lactamase, yielding TX0117c (16–18). The TX0117 and TX0117c MSSA strain pair have been extensively studied in various *in vitro* models and in *in vivo* rat endocarditis models to better understand the comparative efficacy of different antibiotics against MSSA exhibiting the beta-lactamase-mediated cefazolin inoculum effect and against an isogenic MSSA that has been cured of its beta-lactamase (19, 20).

**FIG 1:**
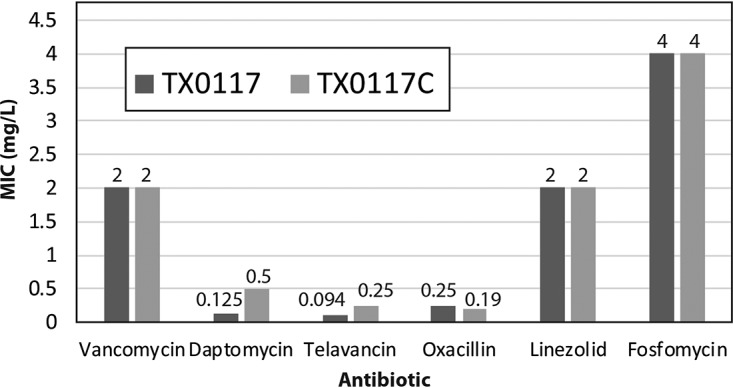
Susceptibilities (MIC, mg/liter) of different antibiotics against TX0117 and beta-lactamase-cured derivative TX0117c, determined by Etest.

Our evaluation of TX0117 and TX0117c showed subtle but consistent increased resistance to cationic antimicrobial peptides in strain TX0117c compared to that in the TX0117 parent strain (Fig. 1), leading us to hypothesize that in addition to curing the strain of beta-lactamase, novobiocin and heat treatment may have additionally coselected previously uncharacterized mutations in TX0117c. To investigate these mutations, we mapped short reads from the TX0117c genome to our newly sequenced and assembled TX0117 genome using breseq version 0.31.0 (option breseq -r TX0117_reference.gbk TX0117c_R1.fastq TX0117c_R2.fasta) (21).

The growth-improved clones were isolated and grown in M9 minimal medium supplemented with 4 g/liter glucose. Cells were then harvested while in exponential growth, and genomic DNA was extracted using a KingFisher Flex purification system previously validated for the high-throughput platform mentioned below (22). Shotgun metagenomic sequencing libraries were prepared using a miniaturized version of the HyperPlus Illumina-compatible library prep kit (Kapa Biosystems). DNA extracts were normalized to 5 ng total input per sample using an Echo 550 acoustic liquid-handling robot (Labcyte, Inc.), and 1/10 scale enzymatic fragmentation, end repair, and adapter ligation reactions were carried out using a Mosquito high-throughput sequencing (HTS) liquid-handling robot (TTP Labtech, Inc.). Sequencing adapters were based on the iTru protocol (23), in which short universal adapter stubs are ligated first, and then sample-specific barcoded sequences are added in a subsequent PCR step. Amplified and barcoded libraries were then quantified using a PicoGreen assay and pooled in approximately equimolar ratios before being sequenced on an Illumina HiSeq 4000 instrument with a paired-end protocol and read lengths of 150 nucleotides (nt). For all software, default parameters were used throughout, unless otherwise noted. The resulting short reads were checked for quality control using FastQC (version 0.11.5), which showed that 698,669 paired-end reads were produced in the TX0117c sequencing run with 32% GC content, and approximately 710,028 paired-end reads were produced in the TX0117 run with 33% GC content. The short reads were then assembled with Unicycler (version 0.4.2) (24). The draft TX0117 genome consists of 163 contigs and 2.758 Mb in total. The final assembled genome was annotated using Prokka (version 1.12) (25). The genome has 2,562 annotated coding sequences (CDSs), 16 tRNAs, and 4 rRNAs.

Using the breseq mutation prediction pipeline, we identified genes altered from TX0117 to the TX0117c strain (Table 1). In addition to seven deletions corresponding to regions of decreased coverage, six coding mutations were identified, which will be the focus of this initial study. Most noteworthy is the curing of *blaZ*, as previously documented. The major mechanism of penicillin resistance, involving the hydrolysis of the beta-lactam ring, has been attributed to beta-lactamase, which is encoded by the *blaZ* gene (26). Type A beta-lactamases contribute to more efficient inactivation of beta-lactam drugs and therefore correlate to the inoculum effect (27).

**TABLE 1:** Differences in coding regions between TX0117 and TX0117C

Gene	Description	Mutation	Annotation
*TX0117_02137*–*TX0117_02167*	*lytN_3*, *atl_4*	Δ25,714 bp	
*TX0117_02458*–*TX0117_02470*	*dnaC_2*	Δ6,772 bp	
*TX0117_02498*–*TX0117_02505*		Δ4,321 bp	
*TX0117_02518*–*TX0117_02522*		Δ2,700 bp	
*TX0117_02535*–*TX0117_02538*		Δ1,460 bp	
*intQ*	*intQ*	Δ1,166 bp	
*xis*–T*X0117_02547*	*xis*	Δ928 bp	
*blaZ*←	Beta-lactamase	(T)_8→7_	Coding (92/846 nt)
*relA*→	GTP pyrophosphokinase	C → T	Q657* (CAA → TAA) (86% truncation)
*TX0117_02559*→	Hypothetical protein	T → A	N24K (AAT → AAA)
*TX0117_01069*←	Hyaluronate lyase	T → C	N476S (AAT → AGT)
		T → C	I471V (ATC → GTC)
		T → C	D457G (GAC → GGC)
		T → G	S450R (AGT → CGT)
		T → G	K271T (AAA → ACA)
		A → T	L262M (TTG → ATG)
		C → T	V254I (GTT → ATT)
*mnaA_1*→	UDP-*N*-acetylglucosamine 2-epimerase	C → T	P149S (CCA → TCA)
*TX0117_02434*←	65-kDa membrane protein	A → T	N181K (AAT → AAA)
		C → T	S177N (AGC → AAC)

TX0117c also displayed a truncation of *relA*, the GTP pyrophosphokinase involved in the stress response. *relA* encodes the RELA protein, which most commonly binds NFKB1 to form a NF-kappa-B transcription factor, activated downstream by processes such as inflammation, tumorigenesis, and differentiation (28). Also mutated, via substitution, is the *mnaA_1* gene. It encodes a UDP-*N*-acetylglucosamine 2-epimerase responsible for converting UDP-GlcNAc into UDP-*N*-acetyl mannosamine, which is then oxidized in the formation of teichoic acids (29). Teichoic acids bind to either peptidoglycans or cytoplasmic membranes and dictate functions from cellular shape to pathogenesis. They have been proven necessary for beta-lactam resistance in methicillin-resistant Staphylococcus aureus (MRSA) (30) and have been shown to control bacteria susceptibility to antimicrobial peptides and cationic antibiotics (31, 32). Additional studies will be needed to examine the role of *relA* and *mnaA* in S. aureus susceptibility to cationic antimicrobial peptides.

### Data availability.

This whole-genome sequencing project has been deposited in NCBI GenBank under the accession no. VSSN00000000, and the Illumina short read data for TX0117 and TX0117c have been deposited in the SRA under the accession no. SAMN12622398 and SAMN12622402, respectively.
